# The Plastic Crisis, Human Dignity, and Care for our Common Home

**DOI:** 10.5334/aogh.4100

**Published:** 2023-03-21

**Authors:** Cardinal Michael Czerny

**Affiliations:** 1 S.J., Vatican Dicastery for Promoting Integral Human Development, VA

**Keywords:** plastic pollution, environmental health, common good, common home

Two images come to mind as I read the powerful report of the Minderoo-Monaco Commission on Plastics and Human Health in this issue of *Annals of Global Health* [1]. First, I see an aisle in a supermarket in a high-income country full of thousands of wonderfully fresh fruits and vegetables, all of them packaged in gleaming plastic. Then I switch to a picture of a beach in a poor country in Asia, Africa, or Latin America littered with countless thousands of plastic containers, some bearing the same brand names as the items in the supermarket. And on that beach, I see a young mother and her child picking through the trash looking for things that they can recover and recycle to earn the $2 they need to survive for another day.

These two pictures are indeed connected. It is vital to approach the question of plastic and health within a single framework. In the wake of Covid-19, we realize that if the planet is not healthy, we cannot be healthy, and vice versa. Life flourishes only if the ecosystems that sustain human and all biotic forms of life are healthy, and they will be healthy only if people take care of them. Plastic pollution threatens the environment, our health, and that of future generations.

The present report from the Minderoo-Monaco Commission on Plastics and Human Health adopts a comprehensive approach to the impact of plastic on human and planetary welfare. In exhaustive detail, the study documents current patterns of plastic production, use and disposal. Plastics are responsible for huge, growing and deeply unjust damages. The harms occur at every stage of the plastic life cycle, from extraction of the coal, oil, and gas that are plastics’ main feedstocks, through production and use, and on to disposal. They include visible damage such as beach litter and contaminated mid-ocean gyres as well as silent injuries to children’s brains and women’s reproductive organs. They disproportionately affect the world’s poorest and marginalized people. This Commission describes an entire archipelago of plastic-associated damages that for too long have been hiding in plain sight.

Current patterns of plastic production are driven by a linear economic system that focuses almost exclusively on short-term economic gain as measured by Gross Domestic Product (GDP) [2]. This paradigm takes little cognizance of human rights, intergenerational equity, or social justice. It views human life and the earth as infinitely abundant and readily expendable resources, with little heed to the consequences of unlimited exploitation. One result is a 230-fold increase in global plastic production since 1950, and this is projected to triple by 2050 [3].

The time has come to recognize that plastics is not an isolated problem. Like climate change, air pollution, biodiversity loss, ocean acidification, grinding poverty, and escalating inequality, it is one example of humanity’s reckless strip-mining of the earth’s resources, mortgaging the future of coming generations of all living species for the sake of short-term, narrowly-measured economic gain [4]. Since nearly all plastic comes from fossil fuels, whose use is already exacerbating the climate crisis, more fundamental solutions are needed that include but go beyond one-off remedies such as bans on single-use plastics, reduction in the complexity and toxicity of plastic, Extended Producer Responsibility, and the obligatory recovery of plastic waste. Over 90% of plastic waste is not recycled and ends up in landfills or accumulates in the environment at large [5]. More durable solutions will need to be based on lasting realignment of humanity’s relationship with nature and of people’s relationships with one another. But since plastics are not only damaging but also very useful, the voices of vulnerable people, who are disproportionally harmed by them, need to be included in the negotiations of effective policies and regulations governing our use of plastic. I am thinking of waste pickers, “fence line” community residents and workers, breathers of air, and drinkers of water.

In his 2015 encyclical *Laudato si*’, Pope Francis argues that climate change, pollution and biodiversity loss are not only environmental but also moral and ethical challenges [6]. If strategies for protecting the planet are to be effective, they must go beyond environmental remediation. They must also benefit otherwise disadvantaged people, advance social justice and incorporate “a preferential option for the poor.” Francis terms this holistic approach to planetary restoration “Integral Ecology.”

Integral Ecology is a powerful concept. It acknowledges the essential links between humans and the environment. It moves ecological thinking beyond narrowly “green” concerns and puts people squarely in the picture. Integral Ecology is rooted in Francis’s view that the earth is a shared inheritance received from the Creator, a “Common Home” whose good things are meant for everyone and whose responsibilities must be fairly shared.

To realize this vision of a healthier, sustainable, and more just world, two things are needed. Each person, especially those who control disproportionate shares of the earth’s resources, must embrace changes to our behavior. We need to undergo an “ecological conversion,” as Pope Francis expresses it, to embrace a new approach “that will transform our way of living in the world, our lifestyles, our relationship with the Earth’s resources, and generally how we look at humanity and living life” [6].

Societies, governments, and international organizations must undergo their own ecological conversion. Following the lead of their citizens including the poor, they must redirect the enormous power of the multinational corporations that are willy-nilly driving the global plastics crisis and related threats to human and planetary health. A small number of vertically integrated corporations extract most of the world’s carbon, produce most of the world’s fossil fuels, and manufacture most of the world’s plastics [7]. They and their consumers are responsible for the harms discussed in this Commission report [1]. In most countries, these corporations feel bound only to return profit to their shareholders and great benefits to their managers. They are rarely under any legal obligation to treat the earth as a “shared inheritance” and therefore to extract only within a sustainable circular system. Naturally, they resist regulations. Immorally, they corrupt governments. Nearly omnipotent, they blithely continue to pollute. This cannot go on. It is not fair. It must change.
